# Dynamical compensation and structural identifiability of biological models: Analysis, implications, and reconciliation

**DOI:** 10.1371/journal.pcbi.1005878

**Published:** 2017-11-29

**Authors:** Alejandro F. Villaverde, Julio R. Banga

**Affiliations:** Bioprocess Engineering Group, IIM-CSIC, Vigo, Spain; University of Washington, UNITED STATES

## Abstract

The concept of dynamical compensation has been recently introduced to describe the ability of a biological system to keep its output dynamics unchanged in the face of varying parameters. However, the original definition of dynamical compensation amounts to lack of structural identifiability. This is relevant if model parameters need to be estimated, as is often the case in biological modelling. Care should we taken when using an unidentifiable model to extract biological insight: the estimated values of structurally unidentifiable parameters are meaningless, and model predictions about unmeasured state variables can be wrong. Taking this into account, we explore alternative definitions of dynamical compensation that do not necessarily imply structural unidentifiability. Accordingly, we show different ways in which a model can be made identifiable while exhibiting dynamical compensation. Our analyses enable the use of the new concept of dynamical compensation in the context of parameter identification, and reconcile it with the desirable property of structural identifiability.

## Author summary

A robust behaviour is a desirable feature in many biological systems. The study of mechanisms capable of maintaining the transient response unchanged despite environmental disturbances has recently motivated the introduction of a new concept: Dynamical Compensation (DC). However, the original definition of DC with respect to a parameter amounts to structural unidentifiability of that parameter, which means that it cannot be estimated by measuring the model output. Since most biological models have unknown parameters that need to be estimated, DC can be considered a negative property for the purpose of model identification. In this paper we reconcile these two conflicting views by proposing a new definition of DC that captures its intended biological meaning (i.e. robustness, which should be a systemic property, intrinsic to the dynamics) while making it distinct from structural unidentifiability (which is a modelling property that depends on decisions made by the modeller, such as the choice of model outputs or unknown parameters, and on experimental constraints). Our definition enables a model to have DC with respect to a structurally identifiable parameter, thus increasing the applicability of the concept.

## Introduction

Some biological systems are capable of maintaining an approximatively constant output despite environmental fluctuations. It has long been accepted that negative feedback plays a central role in biological phenomena such as homeostasis. Feedback mechanisms are capable of rendering a system robust to a wide range of external disturbances. The ability to keep a constant *steady state* has been called exact adaptation, a feature that is known to be achievable with integral feedback [1–5]. The ability of preserving not only the steady state, but also the *transient response* (i.e. the dynamic behaviour) has been less studied and, despite recent contributions [6, 7], the mechanisms that make it possible are still less well understood.

Recently, Karin et al. [6] addressed the problem of finding mechanisms that allowed to maintain the transient response unchanged in the face of environmental disturbances. To describe this phenomenon they coined the term *dynamical compensation* with respect to a parameter, which they defined as the property that the output of a system does not depend on the value of that parameter. According to this definition, dynamical compensation amounts to the parameter being *structurally unidentifiable*. Structural identifiability is a mathematical property originally introduced by Bellman and Åström [8]. If a parameter is structurally unidentifiable, it cannot be determined from experiments because there is an infinite number of values that yield the same model output. For example, symmetric expressions such as *A* = *p*_1_ × *p*_2_ or *B* = *p*_1_ + *p*_2_ yield the same result if the values of *p*_1_ and *p*_2_ are exchanged, so it is not possible to infer *p*_1_ and *p*_2_ by measuring functions of *A* and/or *B*. In such case parameters *p*_1_ and *p*_2_ are called structurally unidentifiable; a model containing structurally unidentifiable parameters is also termed structurally unidentifiable. While in the aforementioned examples structural unidentifiability is apparent, in practice it can be very difficult to detect such situation, even for small models, and many methodologies have been developed for this purpose, as reviewed e.g. in [9–13]. Structurally unidentifiable parameters pose several problems. Their estimated values are biologically meaningless [14], and the use of a structurally unidentifiable model for predicting the time course of system variables that cannot be directly measured can produce wrong results [15]. This means that the usefulness of a model for obtaining biological insight can be compromised if its structural identifiability is not analysed. In recent years the importance of performing such analyses has been highlighted when modelling e.g. HIV infection [16], diabetes [14], infarction [17], or cancer therapeutics [18]. Therefore, the correspondence between dynamical compensation and structural unidentifiability is relevant in realistic situations, in which the parameters of interest can be unknown.

The equivalence between the original definition of dynamical compensation and structural unidentifiability was originally noted in [19, 20]. In one of those papers [19], Sontag drew an additional connection between dynamical compensation and system equivalence, and showed that a related property, fold-change detection (FCD) or input symmetry invariance, is a particular case of the same phenomenon. In this paper we begin by illustrating the correspondence between structural unidentifiability and the original definition of dynamical compensation, which we refer to as DC1, using the four case studies presented by Karin et al. [6]. Then, as a new contribution, we suggest a more complete definition (DC2) drawing from ideas implicit in the original publication [6]. Furthermore, given that structural identifiability is a desirable property for system identification, we enquire whether it is possible to reconcile the concept of dynamical compensation with it. We provide a positive answer by suggesting an alternative definition of dynamical compensation (DC-Id) which does not necessarily imply lack of structural identifiability, and preserves the intended meaning of the DC concept. Using for illustrative purposes one of the circuits proposed by Karin et al. [6], we explore different modelling choices and show how they affect the identifiability of the model. We also compare our proposal with a different one suggested in a note by Karin, Alon, and Sontag [21], which provides an alternative definition of dynamical compensation (called DC3 in the present paper) and analyses the aforementioned circuit. Finally, we discuss the implications of structural unidentifiability and show different ways in which it can be avoided, leading to identifiable models which may or may not exhibit the proposed definition of dynamical compensation (DC-Id).

## Results

### The original definition of dynamical compensation (DC1) is equivalent to structural unidentifiability

Karin et al. [6] introduced the concept of dynamical compensation to describe a design principle that provides robustness to physiological circuits. The original definition of dynamical compensation, which we refer to as “DC1”, is as follows:

#### DC1 definition of dynamical compensation

“Consider a system with an input *u*(*t*) and an output *y*(*t*, *s*) such that *s* > 0 is a parameter of the system. The system is initially at steady state with *u*(0) = 0. Dynamical compensation (DC) with respect to *s* is that for any input *u*(*t*) and any (constant) *s* the output of the system *y*(*t*, *s*) does not depend on *s*. That is, for any *s*_1_, *s*_2_ and for any time-dependent input *u*(*t*), *y*(*t*, *s*_1_) = *y*(*t*, *s*_2_)”. [6]

The DC1 property is similar to the classic definition of structural unidentifiability. A parameter is structurally unidentifiable if it cannot be determined from any experiment, because there are different parameter values that produce the same observations. A model is termed structurally unidentifiable if it has one or more structurally unidentifiable parameters. Using the same notation as in the DC1 definition, structural unidentifiability can be defined as follows [22, 23]:

#### Structural unidentifiability of a parameter

A parameter *s* is structurally identifiable if it can be uniquely determined from the system output, that is, if for any *s*_1_, *s*_2_ it holds that *y*(*t*, *s*_1_) = *y*(*t*, *s*_2_) ⇔ *s*_1_ = *s*_2_. If this relationship does not hold for any *u*(*t*), even in a small neighbourhood of *s*, the parameter is structurally unidentifiable.

**Fig 1:**
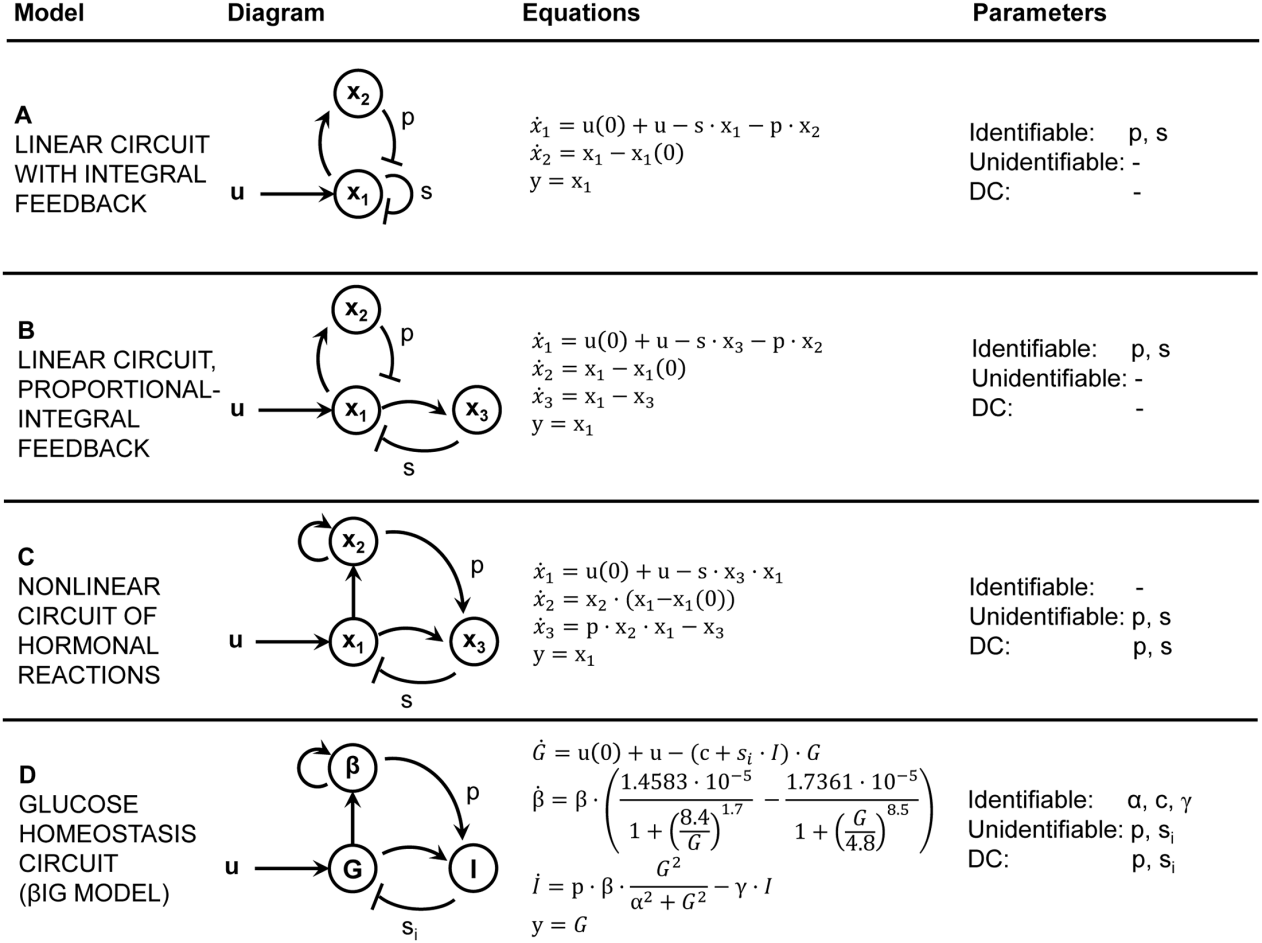
Physiological systems used as case studies. The four circuits shown here represent deterministic dynamic models described by ordinary differential equations (ODEs) [6]. (A) Linear circuit, integral feedback. (B) Linear circuit, proportional-integral feedback. (C) Nonlinear circuit of hormonal reactions. (D) Glucose homeostasis circuit, also known as *β*IG model. Parameters *p* and *s* (or *s*_*i*_) represent gain constants of the feedback loops present in the circuits.

Thus, DC1 can be considered as a particular case of structural unidentifiability of a parameter, with the additional requirement that the system is initially at steady state and with zero input. Here we demonstrate this equivalence by interrogating the structural identifiability of the parameters of the four case studies presented by Karin et al. [6]. They are the circuits shown in Fig 1, which model possible regulatory mechanisms and are described by ordinary differential equations (ODEs).

The model depicted in Fig 1A is a linear system with integral feedback on the output variable, *y*. It corresponds to the circuit of Fig 1B from [6]. The system depicted in Fig 1B is also linear, but has proportional-integral feedback and includes an additional state variable. It corresponds to the circuit of Fig 1C from [6]. The nonlinear model of hormonal reactions shown in Fig 1C corresponds to the one in Fig 1D from [6]. Finally, the fourth case study (Fig 1D) has the same high-level diagram as the previous one (Fig 1C); however, the detailed dynamics are different. This circuit is known as the “*β*IG model” due to its three states (*β*, I, and G). It describes a glucose homeostasis mechanism where *β* stands for the beta-cell functional mass, *I* for insulin, and *G* for glucose. The presence of terms such as (8.4/*G*)^1.7^ makes this system non-rational, which complicates its analysis. We analysed the structural identifiability of these models using the STRIKE-GOLDD tool [24] described in the Methods Section. We obtained that the parameters (*p*, *s*) of the two models exhibiting dynamical compensation—i.e. the hormone circuit of Fig 1C and the *β*IG model of Fig 1D—are structurally unidentifiable, while in the models that have exact adaptation but not dynamical compensation—i.e. the ones shown in Fig 1A and 1B—those parameters are identifiable. Likewise, parameters (*α*, *c*, *γ*) in the *β*IG model are structurally identifiable, and the model does not have dynamical compensation with respect to them.

### The meaning of dynamical compensation and an alternative definition (DC2)

As explained above, the DC1 definition explicitly provided by Karin et al. [6] does not mention certain aspects whose omission can lead to confusion, and in fact, it can be considered as a rephrasing of the structural unidentifiability property [19, 20]. However, the concept of dynamical compensation was not introduced with the aim of describing the same issue as structural unidentifiability. Instead, it was purported to describe a different phenomenon, specifically relevant for the regulation of physiological systems. To clarify the intended meaning of dynamical compensation in the context it was proposed, we use the *β*IG model of Fig 1D as an example. This model describes a glucose homeostasis mechanism where *β* stands for the beta-cell functional mass, *I* for insulin, and *G* for glucose.

**Fig 2:**
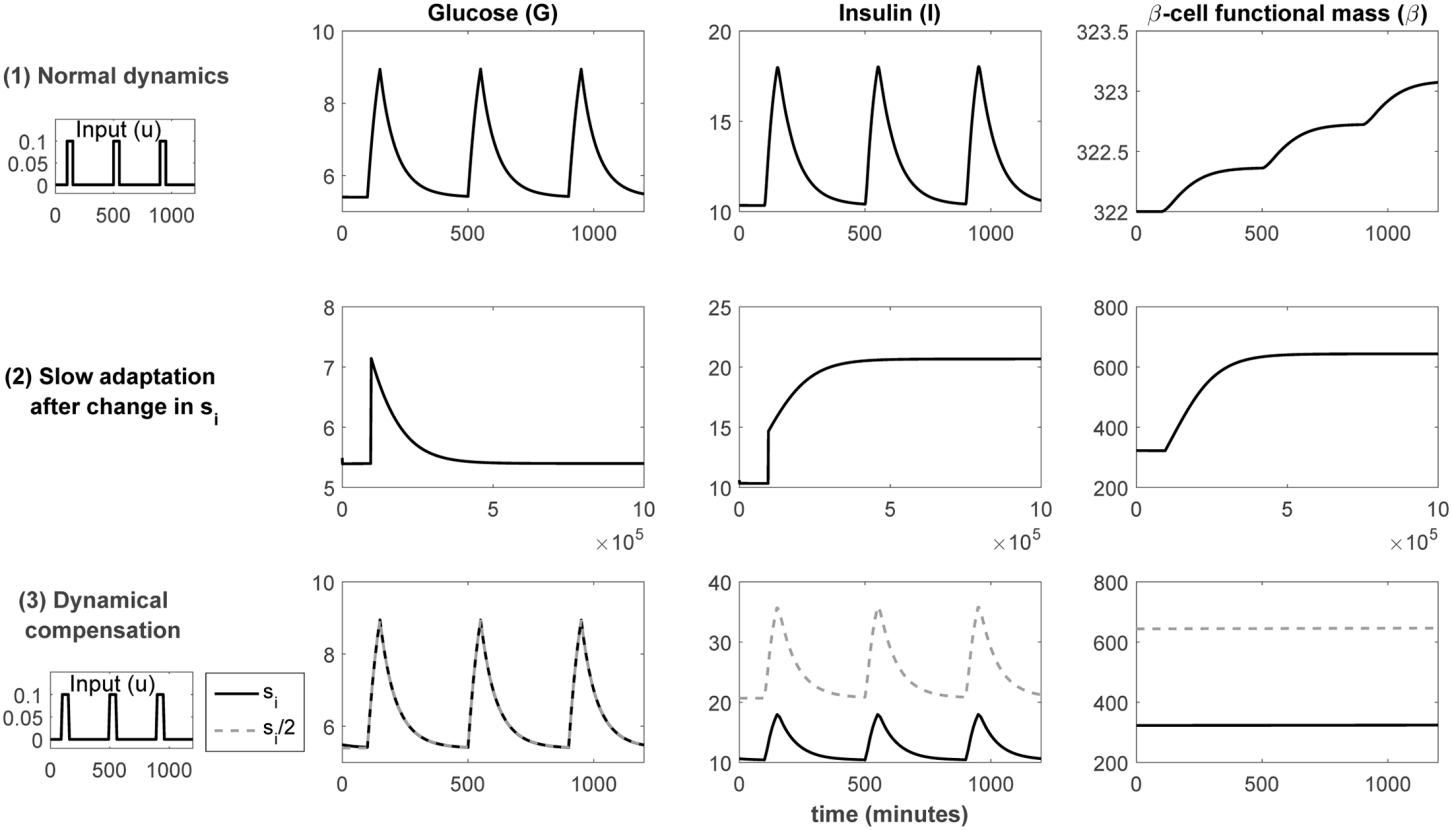
Illustration of the phenomenon of dynamical compensation in a physiological circuit. The upper row shows the normal behaviour of the *β*IG model for a given value of the *s*_*i*_ parameter. The second row shows the evolution of the steady state after a change in the value of *s*_*i*_. After a long adaptation period, which can take months, a new steady state is reached (note that in the plots in this row no external inputs are applied, for clarity of visualization; if they were, periodic peaks similar to the ones in the first and third rows would appear superimposed on the plotted curves). Then, as shown in the third row, the response of the glucose concentration for the new parameter value is the same as the initial one (this does not happen for insulin and *β*-cell mass).

The time evolution of its three states in typical scenarios is shown in Fig 2. The first row describes the behaviour after a series of pulses in glucose, where a pulse corresponds to an external input of glucose resulting from a meal. We consider a typical scenario of three meals, with roughly six hours between them. Both glucose and insulin concentrations reach peaks shortly after the meals, and in a few hours they return to their normal levels (steady state).

The second row describes what happens if the value of a parameter, insulin sensitivity (*s*_*i*_), is changed. Specifically, the figure represents the case in which $s_{i}^{new}=0.5s_{i}^{old}$. For ease of visualization no external pulses are applied in this simulation, so the plots in this row show the evolution of the system with zero input. There is a slow adaptation of the system’s steady state, which can take months, as seen in the figure. After this period the system has adapted to a new steady state: for glucose concentration it remains the same as the initial one (exact adaptation), while the values of insulin concentration and *β*-cell mass are doubled.

The third row illustrates the phenomenon of DC itself: after the adaptation to a new steady state has occurred, the output of the system (from the new steady state, and with the new value of insulin sensitivity) as a response to a pulse in glucose is *the same as before the parameter change* (from the old steady state and the old parameter value). Note that only the glucose dynamics remains unchanged; for insulin and *β*-cell mass there is a scaling.

In light of this behaviour, the following alternative definition of dynamical compensation (DC2) may be deduced from a detailed reading of the original paper by Karin et al.:

#### DC2 definition of dynamical compensation

“Consider a model of a dynamical system with an input *u*(*t*), a set of states *x*(*t*), and an output *y*(*t*, *s*)), such that *s* > 0 is a *known* parameter. The system is initially at a steady state *x*(0) = *ξ* with *u*(0) = 0. The dependence of the output on the initial steady state is denoted by *y*(*t*, *s*, *ξ*). Dynamical compensation (DC) with respect to *s* is that for any parameter values *s*_1_, *s*_2_, for any time-dependent input *u*(*t*), and for two different initial steady states, *ξ*_1_ ≠ *ξ*_2_, the output of the system does not depend on *s*, that is, *y*(*t*, *s*_1_, *ξ*_1_) = *y*(*t*, *s*_2_, *ξ*_2_).”

**Fig 3:**
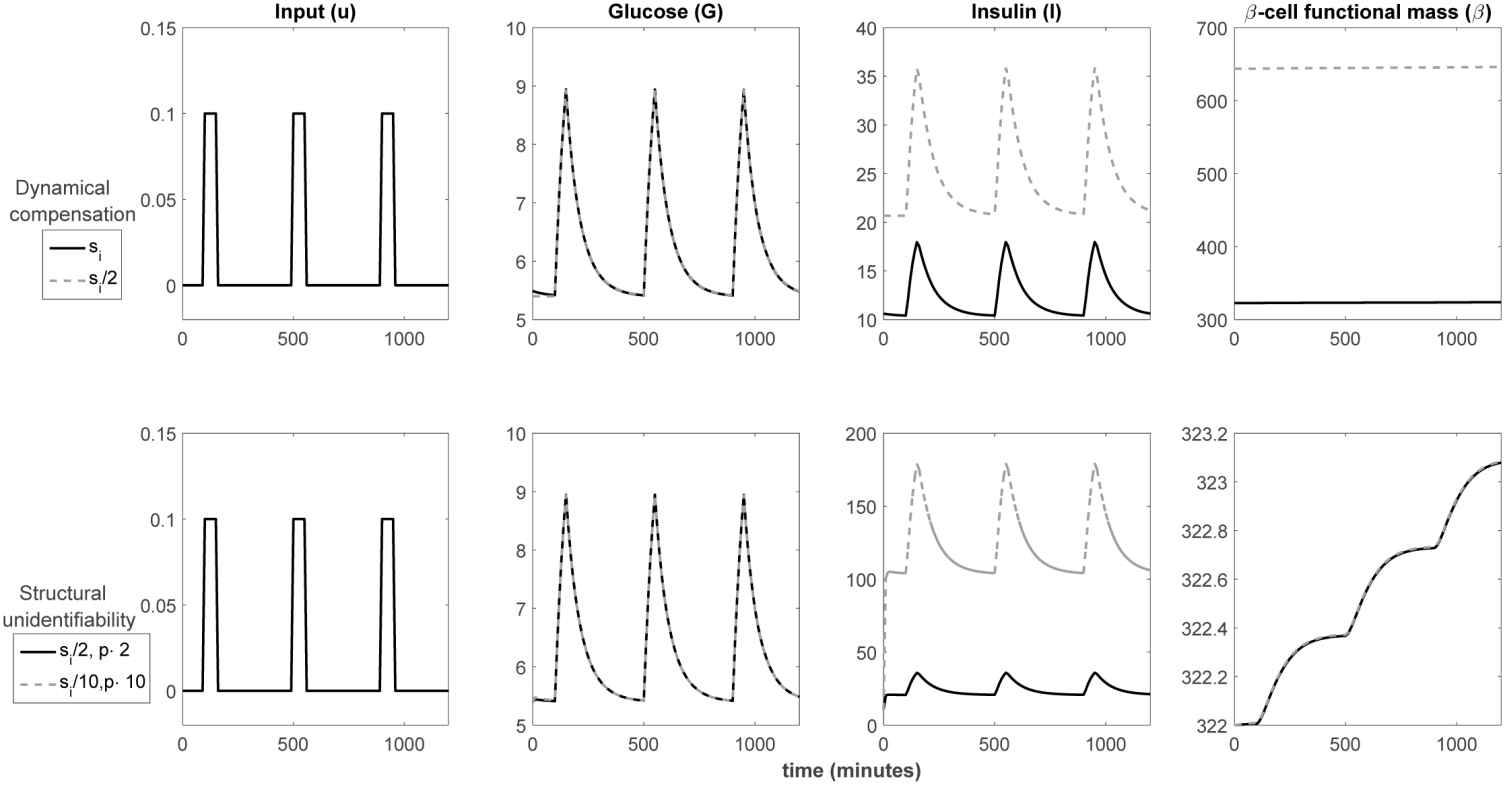
Dynamical compensation and structural unidentifiability in the *β*IG model. The first row reproduces the last row of Fig 2 and illustrates the phenomenon of dynamical compensation: after the system has adapted to the new value of *s*_*i*_, the time-evolution of the glucose concentration (G) for the new value of (*s*_*i*_/2) is the same as it was with the old value before adaptation (*s*_*i*_). The second row illustrates the phenomenon of structural unidentifiability: without the need for any adaptation, the time-evolution of the glucose concentration (G) is the same for any value of the parameter *s*_*i*_, as long as any deviations from the original value are compensated by changes in the parameter *p*. Note that, since the upper and lower plots of G are identical, if glucose is the only measured quantity both phenomena cannot be distinguished. However, the behaviour of the other state variables (I, *β*) can be very different, as can be noticed from the third and fourth columns.

We have tried to keep this new definition, DC2, as similar as possible to DC1, and used the same notation. The DC2 definition of dynamical compensation makes it different from structural unidentifiability. However, it assumes that the parameter of interest is known, which may not be the case in practice. Why is this requirement of known parameters necessary? Let us illustrate this point with Fig 3. It shows in its first row the aforementioned example of dynamical compensation, which has already been discussed (the first row in Fig 3 is the same as the third one in Fig 2). Now, let us assume that the parameters *p*, *s*_*i*_ are unknown, to see the role played by structural unidentifiability. This is shown in the second row of Fig 3. It can be seen that different values of *s*_*i*_ result in the same dynamic behaviour of glucose concentration, as long as the change in *s*_*i*_ is compensated by a coordinated change in *p*. If, as suggested by Karin et al. [6], glucose is the only measured variable, *p*, *s*_*i*_ are structurally unidentifiable: their values cannot be determined, because there is an infinite number of possible combinations of values that yield the same output. This can be problematic, because assuming wrong values for *p*, *s*_*i*_ results in incorrect predictions of the concentration of insulin, as can be observed in the third column. In fact, values of *p*, *s*_*i*_ that yield the same curve of glucose can correspond to totally unrealistic curves of insulin. Importantly, since there is only one measured output (glucose), dynamical compensation cannot be distinguished from structural unidentifiability: by looking only at the second column in Fig 3, we cannot distinguish between the first and the second rows. For this reason, “true” dynamical compensation with respect to a parameter can only be claimed if structural unidentifiability of that parameter can be ruled out. This is the reason for enforcing known parameters in the DC2 definition.

It might be argued that, if a parameter is considered *strictly* constant, its value cannot change due to environmental fluctuations, by definition. In such a context, a phenomenon such as the one described by dynamical compensation could still exist: there could be two instances of a model with different parameter values but the same output. However, in that case it would not have the meaning described in this section and intended by its proponents, that is, a compensation resulting from a feedback mechanism.

### Dynamical compensation in realistic scenarios: DC-Id

In reality, biological models almost always have a number of unknown parameters, whose values must be determined before the model can be used in practical applications. In this context the following question naturally arises: how does the behaviour described by the concept of dynamical compensation relate with structural identifiability of the parameters in the model? As we have already mentioned, structural unidentifiability was shown to be equivalent to the original explicit definition of dynamical compensation, or DC1 [19, 20]. The DC2 definition avoids this equivalence by requiring that the parameter is known. Is this, then, the end of the question? Are unknown parameters with dynamical compensation “doomed” to be structurally unidentifiable, thus potentially limiting the biological insight that can be extracted from the models in which they appear?

We claim here that this is not necessarily the case, provided that we reformulate the definition of dynamical compensation. To show this, let us examine in more detail the structural identifiability of a system with dynamical compensation, the *β*IG model of Fig 1D. We analysed the structural identifiability of this model in its original formulation earlier in this paper, showing that, when its five parameters (*p*, *s*_*i*_, *γ*, *c*, *α*) are considered unknown and plasma glucose concentration (G) is the only available measurement, the two parameters that exhibit dynamical compensation (*p*, *s*_*i*_) are unidentifiable, while the remaining three are identifiable.

Let us now see the results of such analysis when we change key aspects of the model, while preserving its dynamics. The two main choices we can play with are: (i) which parameters of the model are considered unknown, and therefore need to be estimated; and (ii) which measurements are possible. Regarding the first choice (i), we analyse not only the five-parameter case considered by Karin et al., but also other representative scenarios: when the unknown parameters are {*s*_*i*_, *γ*, *c*, *α*} (i.e., all but *p*), when they are {*p*, *s*_*i*_}, and when there is only one unknown, *s*_*i*_. The second choice (ii) defines the output function of the model. While in general the output can be any function of the states, typically it consists of a subset of the states. In the version of the *β*IG model used by Karin et al. [6] the only measured variable was glucose concentration (G). Here we consider all the possibilities, to assess the consequences of measuring every possible combination of the three state variables of the model: glucose (G) and insulin (I) concentrations, and beta-cell mass (*β*). The set of 28 alternative model configurations and the corresponding results of the structural identifiability analysis are summarized in Table 1. It can be noticed that there is substantial variability in the identifiability results depending on the modelling choices, despite the fact that the dynamic behaviour of the system is the same in all cases.

**Table 1:** Structurally unidentifiable parameters for different configurations of the *β*IG model. Each table entry in the four rightmost columns shows the structurally unidentifiable parameters for a given choice of measured outputs (different rows) and parameters considered unknown (different columns). Four representative choices of parameters are studied: (i) with all the model parameters {*α*, *γ*, *c*, *p*, *s*_*i*_} considered unknown, (ii) with the two parameters {*p*, *s*_*i*_} that may exhibit dynamical compensation considered unknown, (iii) with all but *p* unknown, and (iv) with only one parameter, *s*_*i*_, considered unknown.

Measured outputs	Unknown parameters			
	{*α*, *γ*, *c*, *p*, *s*_*i*_}	{*p*, *s*_*i*_}	{*α*, *γ*, *c*, *s*_*i*_}	*s*_*i*_
G	{*p*, *s*_*i*_}	{*p*, *s*_*i*_}	*s*_*i*_	*s*_*i*_
*β*	{*p*, *s*_*i*_}	{*p*, *s*_*i*_}	-	-
I	*p*	*p*	-	-
G, I	*p*	*p*	-	-
G, *β*	{*p*, *s*_*i*_}	{*p*, *s*_*i*_}	-	-
I, *β*	-	-	-	-
*β*, I, G	-	-	-	-

Let us now see how the different configurations in Table 1 affect dynamical compensation. It should be noted that both the original definition of dynamical compensation (DC1) and the second one (DC2) consider single-output models. Specifically, Karin et al. demonstrated that the *β*IG model has dynamical compensation in glucose concentration (G) with respect to the {*p*, *s*_*i*_} parameters. As can be seen in the first row of Table 1, both parameters are structurally unidentifiable when G is the only output. To break this correspondence between dynamical compensation and structural unidentifiability we might interpret the “output” in the DC definition to be multi-dimensional. Indeed, if we could measure the three state variables we would make {*p*, *s*_*i*_} identifiable. However, by doing so we would also destroy the dynamical compensation property, because there is no DC for *β* and I, as seen in Figs 2 and 3. Thus, additional precisions should be incorporated into our working definition of dynamical compensation in order to make it describe a meaningful systemic property without being equivalent to structural unidentifiability. In light of this, we propose the following definition of dynamical compensation, which we call DC-Id:

#### DC-Id definition of dynamical compensation

“Consider a nonlinear time-invariant dynamic system modelled as a structure *M* with the following equations:

$$\begin{array}{c} M:\{\begin{array}{ccc} \dot{x}\left(t\right) & = & f(x(t),p,u(t))\\ y(t) & = & h(x(t),p)\\ x_{0} & = & \xi (p) \end{array} \end{array}$$ (1)

where *f* and *h* are vector functions, $p\in R^{q}$ is a real-valued vector of parameters, $u\in R^{r}$ is the input vector, $x\in R^{n}$ the state variable vector, and $y\in R^{m}$ the output or observables vector. The parameters *p* can be known or unknown constants. The system is initially at a steady state *ξ*, with *u*(0) = 0. The initial state is denoted as *x*_0_. The dependence of the *i*^*th*^ output *y*_*i*_(*t*) on the initial steady state and on a particular value of a parameter *p*_*i*_ ⊂ *p* can be made explicit by writing it as *y*_*i*_(*t*|*p*_*i*_ = *k*, *x*_0_ = *ξ*). Then, we say that a particular model output *y*_*i*_ ⊂ *y* has dynamical compensation (DC) with respect to a parameter *p*_*i*_ ⊂ *p* if, for any two values of *p*_*i*_ (*k*_1_ and *k*_2_), it holds that

1. if *u*(*t*) = 0, *y*_*i*_(*t* → ∞|*p*_*i*_ = *k*_2_, *x*_0_ = *ξ*_1_) → *y*_*i*_(*t* = 0|*p*_*i*_ = *k*_1_, *x*_0_ = *ξ*_1_) (exact adaptation), and
2. for two different initial steady states (*ξ*_1_ ≠ *ξ*_2_), the output *y*_*i*_ does not depend on *p*_*i*_, that is, *y*_*i*_(*t*|*p*_*i*_ = *k*_1_, *x*_0_ = *ξ*_1_) = *y*_*i*_(*t*|*p*_*i*_ = *k*_2_, *x*_0_ = *ξ*_2_), for any time-dependent input *u*(*t*).”

This new definition effectively distinguishes the phenomenon of dynamical compensation from the structural unidentifiability property, by explicitly acknowledging that it applies to a subset (possibly only one) of the model outputs and to a subset of the parameters. When applied to the different model configurations of Table 1, the DC-Id definition yields that there is indeed dynamical compensation for the G output (glucose concentration) with respect to the {*p*, *s*_*i*_} parameters in all cases, and not only for the unidentifiable ones.

### An alternative proposal by Karin, Alon, and Sontag (DC3)

Shortly after the original DC publication [6], two preprints noting the equivalence between DC and structural unidentifiability were posted: one by Sontag [25], which was later published in this journal [19], and our own [20]. Likewise, a few months later two new preprints appeared independently with the aim of reconciling DC with structural identifiability: the one on which the present paper is based [26] and another one by Karin, Alon, and Sontag [21], which proposed an alternative definition of dynamical compensation that we will call DC3. In the present subsection we comment on the latter one and compare it with our own proposal.

Briefly, the DC3 definition includes two conditions for DC: (i) exact adaptation, and (ii) structural unidentifiability of the parameter of interest. Additionally, one of two alternative conditions must hold: either (iii) identifiability from perturbations, or (iv) identifiability given an additional output function.

While more technical details are provided in [21], the intuition behind DC3 is, in the words of its authors, to “require that while the parameter *p* of a DC model is unidentifiable from measurements of *y* at steady-state, it should be identifiable from other experimental measurements—either from measurements of *y* away from steady-state or from measurements of other system variables.”.

DC3 is clearly a more accurate definition of dynamical compensation than DC1 and DC2. It is better at describing the biological phenomenon of interest, and discusses the relationship between the new property and structural (un)identifiability. It also acknowledges that it is desirable to have an identifiable DC parameter, and suggests ways of making it structurally identifiable. However, there are two main concerns with DC3 and the results provided in [21].

The first one is that the reference to measurements of “other variables” is somewhat confusing because, by definition, the output of a model consists of the quantities that are measured (which are often states, but may sometimes consist of the sum or other functions of the states and parameters) and, since the output function is part of the model structure, changing it by measuring *additional* state variables amounts to having a different model. This makes condition (iv) in this definition problematic, strictly speaking. In this regard, it is also worth mentioning that when DC3 is formalized in [21] the state *x*(*t*) is defined as “an n-dimensional vector of state variables” and the output *y*(*t*) as “the output variable”. The use of different wording for each of them seems to imply that *y* is one-dimensional, which would make this definition not valid for the general case of models with multidimensional outputs.

The second—and arguably more important—issue is that the reference to measurements “away from steady-state” can be misleading. This is discussed in the following paragraphs, where we analyse the results presented in [21] and show that the approach suggested in said paper can lead to incorrect conclusions.

The first result reported in [21] is that conditions (i) and (ii) hold for the *β*IG model, and that (iv) also holds if insulin and *β*-cell mass are measured. Therefore the model has dynamical compensation and is structurally identifiable for the output pair {*I*, *β*}. This agrees with our own results, as shown in Table 1.

This result is followed by another analysis, which concludes that “given *p* we can infer *s* either from either (i) measurements of glucose and insulin at steady state, or (ii) measurements of glucose off steady state. To infer *p* we only require some additional measurement such as beta cell mass” (note that *s*_*i*_ is written as *s* in the quoted text).

The paragraph above contains three claims, of which the first one is correct: given knowledge of *p* and measurements of glucose and insulin, we can indeed infer *s*_*i*_, as reported in Table 1. However, the remaining two claims are incorrect. Let us examine them in more detail.

**Fig 4:**
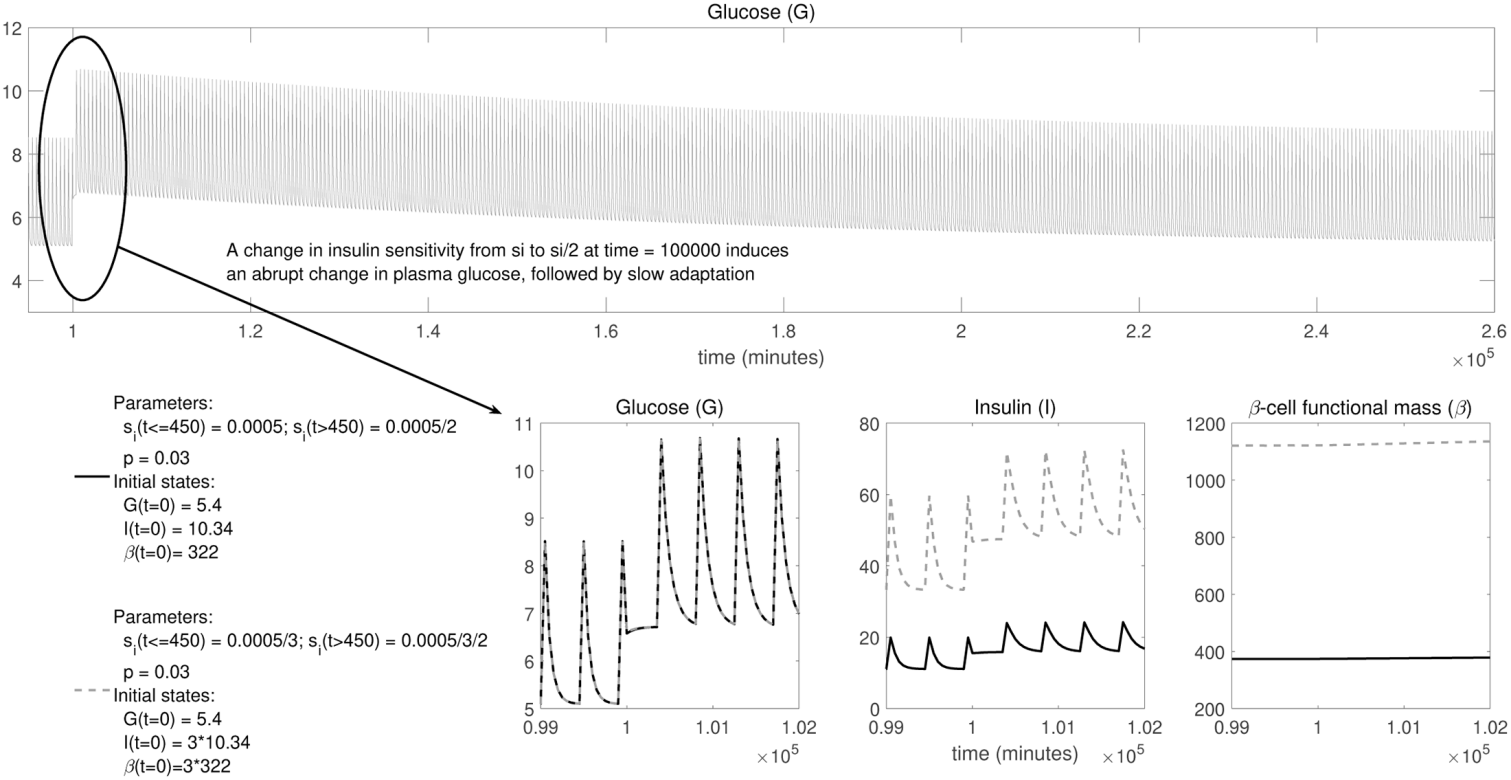
Unidentifiability of *s*_*i*_ from measurements of glucose off steady state. The upper plot shows the time-course of plasma glucose concentration in the *β*IG model in a regime of periodic inputs (pulses) of external glucose from meals, similarly as in Figs 2 and 3. At time 100000 there is a sudden change in the insulin sensitivity parameter, *s*_*i*_, which is halved as a result of external perturbations. This produces an abrupt change in the glucose level, which then undergoes a long period of adaptation until its baseline returns to the original level. The lower plots show in more detail the behaviour of glucose and the other two state variables, insulin and *β*-cell mass, in the hours immediately before and after the change in *s*_*i*_. These plots illustrate that it is not possible to infer the value of the *s*_*i*_ parameter in the *β*IG model by measuring only glucose, even if measurements off steady state are available and *p* is known. This is indicated by the fact that the dynamic time-course of glucose concentration (G) is identical for $s_{i}=s_{i}^{*}$ and $s_{i}=s_{i}^{*}/k$ (left plot in lower row), as long as the initial conditions of the two unmeasured states, insulin (middle plot) and *β*-cell mass (right plot), are multiplied by the same factor *k*. The figure shows results for *k* = 3 and $\left\{s_{i}^{*}=0.0005,p=0.03\right\}$.

One claim is that, given *p*, it is possible to infer *s*_*i*_ from “measurements of glucose off steady state”. It should be noted that most of the measurements that we would usually collect for the *β*IG model (e.g. in a scenario such as the one pictured in the third row of Fig 2, with inputs of glucose from meals) are already *off* steady state (i.e. *dG*(*t*)/*dt* ≠ 0), since the system only returns to steady state a few hours after the external pulse of glucose. However, given the context in which the words “off steady state” are used in [21], we might interpret that Karin et al. are specifically referring to the particular situation that takes place immediately after the insulin sensitivity parameter is changed as a result of a perturbation, which is illustrated in Figure 1 in [21] and in the second row of Fig 2 in the present paper. As explained before, such perturbation instantaneously modifies the system’s steady state, which then goes back to the initial one after a long adaptation period. During this adaptation period the system is transitioning between two different steady states. Figure 1 in [21] shows that, while the glucose curves are identical for (I) *s*_*i*_ *before* adaptation and for (II) *s*_*i*_/2 *after* adaptation, they do not coincide with (III) *s*_*i*_/2 *during* adaptation. For this reason Karin et al. argue that during this period it is possible to identify *s*_*i*_ from glucose measurements. However, this is not true: as shown in Fig 4, the time course of glucose (leftmost plot in the lower row) is the same for a model with *s*_*i*_ and for another with *s*_*i*_/*k*, as long as the initial concentrations of insulin and *β*-cell mass of the second model are multiplied by the same constant *k*. And this holds even if the value of *s*_*i*_ is changed during the course of the experiment, triggering the slow adaptation. What is happening in this case is that, although there is only one unknown parameter (*s*_*i*_), there are also two unmeasured states (*I*, *β*), and it is possible to compensate the variation in model output (*G*) originated from changes in the parameter with coordinated changes in the two unmeasured states. Thus, if only glucose is measured (left plot in Fig 4), it is impossible to distinguish between *s*_*i*_ and *s*_*i*_/*k*—even if we know the value of *p*, which is the same in both cases—and therefore the parameter *s*_*i*_ is structurally unidentifiable. This is in agreement to the results reported in Table 1.

The remaining claim (“To infer *p* we only require some additional measurement such as beta cell mass”) is also incorrect: if not only *s*_*i*_ but also *p* are unknown, and besides glucose we measure also *β*-cell mass, both parameters are unidentifiable even with measurements off steady state. This can be realised by inspecting the lower row of Fig 3, which shows that the time courses of glucose and *β*-cell mass are identical for two different parameter vectors ({*s*_*i*_/2, 2 ⋅ *p*} and {*s*_*i*_/10, 10 ⋅ *p*}), both in and off steady state. More generally, any pair of values {*s*_*i*_/*k*, *k* ⋅ *p*} will yield the same output as a reference vector {*s*_*i*_, *p*}, as long as insulin is not measured. For this model, inferring both {*s*_*i*_, *p*} always requires measuring *at least* *β*-cell mass *and* insulin, as we have shown in this paper (see Table 1).

The cause of the inaccurate claims in [21] is that often times the cause of unidentifiability is the correlation between parameters, or between parameters and state variables. In that case, as happens with the *β*IG model, structural identifiability cannot be determined in a step-wise fashion and intuitive reasoning can lead to misjudgements. Such issues can be circumvented by performing a rigorous structural identifiability analysis and adopting a definition of DC like the one proposed in the present paper, DC-Id.

## Discussion

### Implications of structural unidentifiability

The fact that a model is unidentifiable is important because, after five decades of research, it is now well understood that lack of structural identifiability is the result of choosing an inappropriate model structure for the available measurable variables (or variables that can be directly observed) [23, 27]. When understood in this way, structural unidentifiability can be avoided or surmounted in at least three ways: (i) by reducing the number of parameters or changing their definition, (ii) by increasing the number of measured variables, if possible, or (iii) by determining the unidentifiable parameters in some alternative way, e.g. by direct measurements.

Strategy (i) entails reformulating the model to remove redundant parameters, for example, by grouping several non-identifiable parameters into a single identifiable one. Perhaps the simplest example would be the merging of two parameters that multiply each other into a single one, i.e. *p*_new_ = *p*_1_ × *p*_2_. Such relationships can be revealed systematically by performing a structural identifiability analysis. Some techniques such as COMBOS [28] are explicitly designed for finding identifiable combinations of otherwise unidentifiable parameters, and other methods may also be used for this purpose [29–33].

Strategy (ii) can be illustrated with the “*β*IG” model: if it were possible to measure all its three states instead of only glucose, all the parameters in the *β*IG model would become structurally identifiable. In other words, while the effect on the glucose concentration (G) of a change in *p* can be compensated by changing *s*_*i*_, this does not happen for the insulin concentration (I). Since it may not be realistic to measure *β*-cell mass continuously, another possibility could be to assume it constant (since it changes very little, as can be seen in Fig 3) and use as an estimate of it a single measurement obtained in the past. With this assumption it suffices to monitor the insulin concentration (I) to obtain an identifiable model, as seen in Table 1.

Strategy (iii) was applied for example by Watson et al. [34]. After determining that two parameters in a homeostatic model were structurally unidentifiable, they decided to measure one of them by means of a tracer experiment and to calculate an estimate of the other using a steady state assumption.

Strategies (ii) and (iii) demonstrate how measurements and data can directly inform modelling decisions. More generally, structural identifiability analysis can inform expectations about how precisely a model can be defined, given measurements and data. For example, if the state of a system changes very little when a parameter varies, the system is sometimes said to be *robust* or insensitive to variations in that parameter [27]. Speaking in terms of identifiability, this scenario may be connected to poor *practical identifiability*: although the value of the parameter has some influence on the model output, its effect is too small to allow for its precise determination due to limitations in the information content of the data (regarding quantity and/or quality) [23, 35]. In contrast, when the sensitivity of the model output to a parameter is exactly zero, as implied by DC1, it corresponds to lack of *structural identifiability*. In this case, the value of the parameter has no influence at all on the model output. This situation represents an “unreasonable” elasticity which should not be interpreted as a sign of biological robustness, but as an indication that the parameter is not meaningful. Ideally, it should be removed and the model should be modified, as explained above.

It should be noted that in realistic situations the values of estimated parameters always have some associated uncertainty. Practical identifiability analysis (which is sometimes referred to as numerical identifiability, estimability, or a posteriori identifiability) quantifies the uncertainty that results from limitations in the information content of the data used for calibration [13, 23, 27, 35]. Unlike practical identifiability, dynamical compensation and structural identifiability are both *a priori* concepts, that is, they can be studied before collecting experimental data. In this regard, the uncertainty in parameter estimates does not play a role in dynamical compensation.

As recently stressed by Janzén et al. [36], the danger of inadvertently using a structurally unidentifiable model is that the biological interpretations of its parameters are not valid, which may lead to wrong conclusions; furthermore, any predictions involving unmeasured states “may be meaningless if the parameters directly or indirectly related to those states are unidentifiable” [36]. This fact can be illustrated with the *β*IG model, as seen in the second row of Fig 3: if we try to estimate the *p*, *s*_*i*_ parameters from glucose (G) measurements, we will not be able to recover their true values, because they are structurally unidentifiable: there is an infinite number of combinations of their values that yield the same glucose profile. This, in turn, means that we cannot use the model to predict the time-course of insulin concentration (I), which is an unmeasured state. As seen in the lower plot of the third column, the predictions of insulin can be very different depending on the pair of *p*, *s*_*i*_ values used.

## Conclusions

Given that deficiencies in identifiability may lead to wrong reconstructions of a system’s behaviour, and that parameter identification is an ubiquitous need in biological modelling, it is necessary to assess the structural identifiability of a model before using it to extract insights about the corresponding biological system. The absence of structural identifiability considerations in the paper that introduced dynamical compensation [6] led to an ambiguous definition of the latter concept, which we have termed DC1 in the present manuscript. The fact that DC1 is essentially equivalent to structural unidentifiability when examined from the viewpoint of model identification, as noted in [19, 20], is a source of potential confusion: it opens the door to (i) interpreting as dynamical compensation what might be a case of structural unidentifiability, and to (ii) inadvertently using structurally unidentifiable models.

It is possible to deduce from a detailed reading of the original paper [6] an alternative definition of dynamical compensation (called DC2 in the present manuscript), which removes some ambiguities of DC1. Another alternative definition, which we have called here DC3, was suggested in [21], but its application can be problematic, as shown in this paper. Importantly, neither DC1 nor DC2 are appropriate for realistic modelling scenarios, in which it is necessary to estimate the values of parameters from input-output data. To overcome this limitation we have proposed a modification of the definition of dynamical compensation which can be used in such cases. Our new definition, termed DC-Id, captures the biological meaning of the dynamical compensation phenomenon, which is the invariance of the dynamics of certain state variables of interest with respect to changes in the values of certain parameters. But, additionally, it includes precisions that make it distinct from structural unidentifiability, even in the context of parameter identification—that is, when it is necessary to determine the values of the model parameters. It is thus unambiguous and generally applicable.

We see the discussion held in the present paper and the resulting clarification as an example of the gains that can be obtained by exchanging more notes among the different communities working in biological modelling, which we have advocated elsewhere [37]. Such an exchange of notes increases researchers’ awareness of community-specific knowledge and is useful for avoiding potential misconceptions.

## Methods

### Modelling formalism and mathematical notation

We consider state-space models described by ordinary differential equations (ODEs) of the following general form:

$$\begin{array}{c} M:\{\begin{array}{ccc} \dot{x}\left(t\right) & = & f(x(t),p,u(t))\\ y(t) & = & h(x(t),p)\\ x_{0} & = & x(p) \end{array} \end{array}$$ (2)

Following the usual convention, we use: *x* to refer to state variables, *u* for inputs, *y* for outputs, and *p* for parameters. States, inputs, and outputs are in general time-varying, while parameters are constants (it could also be possible to take into account time-varying parameters, but these are rare in biological models [27]; for an exception, see e.g. the model of glucose turnover by Steele et al. [38]). In Eq (2), *f* and *h* are analytic vector functions of the states and parameters, which are in general nonlinear (linear models are a particular case). For ease of notation we can omit the dependence of *f* and *h* on *p*, and denote initial values of state variables or inputs as *x*_0_ = *x*(0) and *u*_0_ = *u*(0), respectively. We also often drop the time dependence, i.e. we write *x* instead of *x*(*t*), and so on.

We remark that by “model structure” we refer not only to the dynamic equations ($\dot{x}$) but also to the definition of the observation function, or set of measured model outputs (*y*), and the known input variables (*u*). It should also be noted that the model output *y*(*t*) does not take noise into account, since it does not play a role in the concepts discussed in the present paper. Structural identifiability and dynamical compensation are both *a priori* properties, which can be analysed before performing any measurements. Of course, in a realistic parameter estimation scenario it is also necessary to take into account limitations introduced by the quantity and quality of the available data. This is the related topic of *practical* or *numerical* identifiability, which aims at quantifying the uncertainty in the estimated parameter values that results not only from the model structure but also from data limitations, including noise [13, 23, 27, 35].

### Nonlinear observability

Among the existing approaches for structural identifiability (SI) analysis, we adopt one that considers SI as a generalization of observability—the property that allows reconstructing the internal state (*x*) of a model from observations of its outputs (*y*). If a model is observable there is (at least locally) a unique mapping from *y* to *x*, and two different states will lead to two different outputs. Observability is a classic system-theoretic property introduced by Kalman for linear systems, and extended to the nonlinear case by Hermann and Krener [39], among others. It can be studied with a differential geometry approach, as described in the remainder of this subsection. A thorough treatment of this matter can be found in the books by Vidyasagar and Sontag [40, 41].

Observability analysis determines if the mapping from *y* to *x* is locally unique by analysing the expression of *y* = *h*(*x*) and its derivatives. This is done by constructing an observability matrix that defines this mapping, and then calculating its rank. If the matrix is not full rank, the same output can be produced by an infinite number of state vectors, and the system is unobservable. In the nonlinear case, the observability matrix can be built using Lie derivatives. The extended Lie derivative of *h* with respect to *f* is:

$$\begin{array}{c} L_{f}h\left(x\right)=\frac{\partial h(x)}{\partial (x)}f\left(x,u\right)+\sum_{j=0}^{j=\infty }\frac{\partial h(x)}{\partial u^{\left(j\right)}}u^{\left(j+1\right)} \end{array}$$ (3)

where *u*^(*j*)^ and *u*^(*j*+1)^ denote the *i*^*th*^ and (*i* + 1)^*th*^ derivatives of the input, respectively. Higher order Lie derivatives can be recursively calculated from lower order ones as:

$$\begin{array}{c} L_{f}^{i}h\left(x\right)=\frac{\partial L_{f}^{i-1}h\left(x\right)}{\partial x}f\left(x,u\right)+\sum_{j=0}^{j=\infty }\frac{\partial L_{f}^{i-1}h\left(x\right)}{\partial u^{\left(j\right)}}u^{\left(j+1\right)} \end{array}$$ (4)

The nonlinear observability matrix can be written as:

$$\begin{array}{c} O\left(x\right)=(\begin{array}{c} \frac{\partial }{\partial x}h\left(x\right)\\ \frac{\partial }{\partial x}\left(L_{f}h\left(x\right)\right)\\ \frac{\partial }{\partial x}\left(L_{f}^{2}h\left(x\right)\right)\\ \vdots \\ \frac{\partial }{\partial x}\left(L_{f}^{n-1}h\left(x\right)\right) \end{array}) \end{array}$$ (5)

where *n* is the dimension of the state vector *x*. We can now formulate the *Observability Rank Condition (ORC)* as follows: if the system given by Eq (2) satisfies $\text{rank}(O\left(x_{0}\right))=n$, where O is defined by Eq (5), then it is (locally) observable around *x*_0_ [39]. This condition guarantees *local* observability, which means that the state *x*_0_ can be distinguished from any other state in a neighbourhood, but not necessarily from distant states. The distinction between local and global identifiability is usually not relevant in biological applications.

### Structural identifiability as generalized observability

By considering the parameters as state variables with zero dynamics ($\dot{p}=0$), SI analysis can be recast as observability analysis. To this end, we augment the state vector as $\tilde{x}=[x,p]$ and write the generalized observability-identifiability matrix as:

$$\begin{array}{c} O_{I}\left(\tilde{x}\right)=(\begin{array}{c} \frac{\partial }{\partial \tilde{x}}h\left(\tilde{x}\right)\\ \frac{\partial }{\partial \tilde{x}}\left(L_{f}h\left(\tilde{x}\right)\right)\\ \frac{\partial }{\partial \tilde{x}}\left(L_{f}^{2}h\left(\tilde{x}\right)\right)\\ \vdots \\ \frac{\partial }{\partial \tilde{x}}\left(L_{f}^{n+q-1}h\left(\tilde{x}\right)\right) \end{array}) \end{array}$$ (6)

where *n* is the dimension of the state vector *x* and *q* is the dimension of the parameter vector *p*. We can now state a generalized Observability-Identifiability Condition (OIC): if a system satisfies $\text{rank}(O_{I}\left({\tilde{x}}_{0}\right))=n+q$, it is (locally) observable and identifiable around the state ${\tilde{x}}_{0}$.

If $\text{rank}(O_{I}\left({\tilde{x}}_{0}\right))<n+q$, the model contains unidentifiable parameters (and/or unobservable states). It is possible to determine the identifiability of individual parameters because each column in *O*_*I*_ contains the partial derivatives with respect to one parameter (or state). Thus if the matrix rank does not change after removing the *i*^*th*^ column the *i*^*th*^ parameter is not identifiable (if the column corresponds to a state, it is not observable).

### Software

The software used in this paper for analysing structural identifiability is STRIKE-GOLDD (STRuctural Identifiability taKen as Extended-Generalized Observability with Lie Derivatives and Decomposition). It is a methodology and a tool for structural identifiability analysis [24] which can handle nonlinear systems of a very general class, including non-rational ones. At its core is the conception of structural identifiability as a generalization of observability. Since the calculation of $\text{rank}(O_{I}\left({\tilde{x}}_{0}\right))$ can be computationally very demanding, even for models of moderate size, STRIKE-GOLDD includes a number of algorithmic modifications to alleviate its cost. One of them is the construction of the observability-identifiability matrix $O_{I}$ with less than *n* + *q* − 1 derivatives. In certain cases, this reduced matrix can suffice to establish the identifiability of the whole model; in other cases, it can at least report identifiability of a subset of parameters, even if it cannot decide on the rest. Another possibility is to decompose the model in a number of submodels, which have smaller matrices whose rank is easier to compute. More details about these and other procedures included in the methodology can be found in the STRIKE-GOLDD publication [24]. STRIKE-GOLDD is an open source MATLAB toolbox that can be downloaded from https://sites.google.com/site/strikegolddtoolbox/. A more complete description of the tool can be found in its user manual, which is available in the website. All the code (including the STRIKE-GOLDD toolbox) and instructions required for reproducing the results reported in this paper are provided in S1 File.

## Supporting information

S1 File MATLAB code. Compressed ZIP folder including files to reproduce the results reported in this paper. They include two main types of computations: (i) structural identifiability analysis, and (ii) simulation of dynamic models.
(ZIP)
