# Supplementary material for: Dynamical compensation and structural identifiability of biological models: Analysis, implications, and reconciliation
Source: PLoS Comput Biol. 2017 Nov 29;13(11):e1005878. doi: 10.1371/journal.pcbi.1005878 (PMC5724898; doi:10.1371/journal.pcbi.1005878)
Supplement: S1 File — Compressed ZIP folder including files to reproduce the results reported in this paper. They include two main types of computations: (i) structural identifiability analysis, and (ii) simulation of dynamic models. (ZIP) [file pcbi.1005878.s001.zip › Supplementary_Material_MATLAB_code_(S1_ File)_v2/README - Matlab code for reproducing the results.pdf]

MATLAB files of the paper

"Dynamical compensation and structural identifiability of biological models: analysis, implications, and reconciliation"

Alejandro F. Villaverde[1,*] and Julio R. Banga[1]

[1] *Bioprocess Engineering Group, IIM-CSIC, Vigo, Spain*
[*] *afvillaverde@iim.csic.es*

October 30, 2017

This document describes how to reproduce the results presented in the paper "Dynamical compensation and structural identifiability of biological models: analysis, implications, and reconciliation" by Alejandro F. Villaverde and Julio R. Banga [4], by running the MATLAB files provided as supplementary information. The results include two main types of computations: (i) structural identifiability analysis, and (ii) simulation of dynamic models. A preliminary version of this paper, which contains an additional case study, is available in arXiv [3].

Requirements

Running this code requires a MATLAB installation with the Symbolic Math Toolbox (the code has been tested on MATLAB R2015b with Symbolic Math Toolbox 6.3).

1 Structural Identifiability Analysis (SI)

Structural identifiability analysis is performed with the STRIKE-GOLDD toolbox. With this document a version of this toolbox (STRIKE-GOLDD_DC) is provided, which includes scripts for analysing the case studies in [3] and [4].

1.1 Models and files for SI analysis

The four models used as case studies in [4] are taken from [2]; since they correspond to the circuits shown in Figure 1 of [4], we refer to them as 1A, 1B, 1C, and 1D.

Model 1D is known as the "βIG" model. We provide scripts to analyse different versions of this model, depending on which states are considered measured and which parameters are considered known. The set of 28 alternative configurations of model 1D, and the corresponding MATLAB files, are listed in Table 1.

A fifth model, presented by Bolie in [1], is also analysed in [3]. We consider three different versions of it, which we denote as "Bolie A", "Bolie B", and "Bolie C". They are described in [3].

The mat-files containing the definitions of all the models, as well as the m-files used to generate them (whose names start with `z_create...`), are stored in the `models` folder.

	Unknown parameters			
**Outputs**	All ($\{\alpha, \gamma, c, s_i, p\}$)	$\{p, s_i\}$	$\{\alpha, \gamma, c, s_i\}$	s_i
G	1D_out_G_par_agcsp	1D_out_G_par_ps	1D_out_G_par_agcs	1D_out_G_par_s
β	1D_out_B_par_agcsp	1D_out_B_par_ps	1D_out_B_par_agcs	1D_out_B_par_s
I	1D_out_I_par_agcsp	1D_out_I_par_ps	1D_out_I_par_agcs	1D_out_I_par_s
G,I	1D_out_GI_par_agcsp	1D_out_GI_par_ps	1D_out_GI_par_agcs	1D_out_GI_par_s
G,β	1D_out_BG_par_agcsp	1D_out_BG_par_ps	1D_out_BG_par_agcs	1D_out_BG_par_s
I,β	1D_out_BI_par_agcsp	1D_out_BI_par_ps	1D_out_BI_par_agcs	1D_out_BI_par_s
β,I,G	1D_out_BIG_par_agcsp	1D_out_BIG_par_ps	1D_out_BIG_par_agcs	1D_out_BIG_par_s

Table 1: **File names of the different configurations of the βIG model.** The differences between configurations arise from the choice of measured outputs and parameters considered unknown. Four representative choices of parameters are studied: (i) with all the model parameters $\{\alpha, \gamma, c, s_i, p\}$ considered unknown, (ii) with the two parameters $\{p, s_i\}$ that may exhibit dynamical compensation considered unknown, (iii) with all but p unknown, and (iv) with only one parameter, s_i, considered unknown.

1.2 How to perform a SI analysis

To analyse the models you simply need to:

1. Open a MATLAB session and go to the `STRIKE_GOLDD` folder.

2. Execute the file `STRIKE_GOLDD.m` by typing in the MATLAB command window:
   `>> STRIKE_GOLDD`

The two steps above analyse the structural identifiability of the 1A model, because it is the one indicated by default in the `options.m` file. Results are shown in the MATLAB screen and stored as a mat-file in the `results` folder.

To analyse the other examples, you must modify line 6 in the `options.m` file so that it contains the name of the mat-file of the desired model.

NOTE: parameter and state names are encoded differently in the MATLAB scripts and in the original publication. This has to be taken into account when interpreting the results reported by STRIKE-GOLDD. The following naming convention is used:

In the manuscript:	G	β	I	p	s_i	γ	c	α
In the MATLAB scripts:	x_1	x_2	x_3	p_1	p_2	p_3	p_4	p_5

Table 2: **State and parameter names used in the MATLAB scripts.**

2 Simulation results

To illustrate the phenomena of dynamical compensation and structural identifiability, several simulations of the "βIG" model were presented in Figures 2, 3, and 4 of [3]. These figures can be generated by running the m-files `plot_Figures_2_and_3.m` and `plot_Figure_4.m`. To do it, simply open a MATLAB session, go to the directory containing this file, and type:

`>> plot_Figures_2_and_3`

or

`>> plot_Figure_4`

Computational details of these simulations can be learned by inspecting the m-file.

References

[1] Victor W Bolie. Coefficients of normal blood glucose regulation. *Journal of Applied Physiology*, 16(5):783–788, 1961.

[2] Omer Karin, Avital Swisa, Benjamin Glaser, Yuval Dor, and Uri Alon. Dynamical compensation in physiological circuits. *Molecular Systems Biology*, 12(11):886, 2016.

[3] Alejandro F Villaverde and Julio R Banga. Dynamical compensation and structural identifiability: analysis, implications, and reconciliation. *arXiv*, page 1703.08415, 2017.

[4] Alejandro F Villaverde and Julio R Banga. Dynamical compensation and structural identifiability of biological models: analysis, implications, and reconciliation. *PLOS Computational Biology*, 2017.
